# Right tracheal sleeve pneumonectomy with VV-ECMO assistance for non-small cell lung cancer through anterior thoracotomy: a single centre experience

**DOI:** 10.3389/fsurg.2023.1238462

**Published:** 2023-08-03

**Authors:** Valentina Marziali, Giuseppe Mangiameli, Alessandro Crepaldi, Federico Piccioni, Elena Costantini, Enrico Citterio, Alessandro Borbone, Umberto Cariboni

**Affiliations:** ^1^ Division of Thoracic Surgery, IRCCS Humanitas Research Hospital, Milan, Italy; ^2^ Department of Biomedical Sciences, Humanitas University, Milan, Italy; ^3^ Department of Anesthesia and Intensive Care, IRCCS Humanitas Research Hospital, Milan, Italy; ^4^ Department of Cardiac Surgery, IRCCS Humanitas Research Hospital, Milan, Italy

**Keywords:** tracheal sleeve pneumonectomy, NSCLC, ECMO, lung cancer, tracheal surgery

## Abstract

For a long time, non-small cell lung cancer (NSCLC) arising less than 2 cm distal to the carina has been usually considered unresectable and incurable with a radical or extended resection because of surgical technical difficulty and airway management. Recently, the introduction of more advanced surgical techniques, often including the use of extracorporeal life support (ECLS), has allowed us to extend the limits of conventional surgery, increasing the rate of complete surgical resection. ECLS also overcomes the limitation of conventional ventilation during complex tracheo-bronchial reconstruction, avoiding the presence of disturbing lines or tubes that obstruct the operative field during a challenging surgery. In this article, we share our experience in performing right tracheal sleeve pneumonectomy with veno-venous extracorporeal membrane oxygenation (VV-ECMO) in three cases by anterior right thoracotomy, reporting our tips and tricks.

## Introduction

Tumours arising less than 2 cm distal to the carina are still considered a challenge due to surgical technical difficulty and airway management. In the past, they have presented a contraindication to surgical excision. However, in the previous two decades, several studies about extended resection of T4 non-small cell lung cancer (NSCLC) involving the carina have been published, confirming encouraging long-term oncological results (1, 2). Nowadays, Tracheal Sleeve Pneumonectomy (TSP) is the suggested treatment for NSCLC invading the main bronchus or arising at less than 2 cm distal to the carina or at the trachea-bronchial angle with an extension less than three cartilaginous rings of the trachea (3). In 1950, Abbott first describes the procedure of right pneumonectomy with en-bloc resection of the carina with lateral resection of the tracheal wall above the right main bronchus and transverse closure (4), while the first true tracheal sleeve pneumonectomy was published by Gibbon in 1959 (5). Over the following years, different techniques have been proposed, although the mortality rate remained high, ranging between 7.2% and 11.7% (6–9).

Progressive development in the field of anaesthesiology, oncological guidelines, and surgical techniques has improved perioperative outcomes, reducing morbidity and mortality. However, carinal resection and reconstruction still present problems, such as ventilation techniques used during the anastomosis, reconstruction technique, and postoperative complications. Ideal ventilation strategies should ensure several criteria are met, such as sufficient gas exchange and adequate oxygenation when the airway is interrupted, an unobstructed surgical field, and prevention of blood aspiration into the bronchial tree (10). Different methods of intraoperative airway management have been described, including small single-lumen endobronchial tubes, cross-field ventilation, high-frequency jet ventilation (HFJV), intermittent apnoeic ventilation, and extracorporeal membrane oxygenation (ECMO) (11).

ECMO is a more invasive but effective ventilation strategy that provides cardiac or respiratory support without interference with the surgical field (12). The ECMO circuit for an adult patient usually consists of an inflow cannula, a centrifugal pump, a heat exchanger embedded in a membrane oxygenator, and an inflow cannula that transports arterialised blood. Two different types of ECMO are available: veno-venous (VV)- and veno-arterial (VA)-ECMO.

Used most frequently in the treatment of severe respiratory failure, VV-ECMO has both the inflow and outflow cannula placed in the patient's venous circulation. VA-ECMO allows for full cardiac or pulmonary support. It can be used to provide vital support to the organs, temporary circulatory support, and/or relief to the heart during myocardial recovery. It can also be a bridge to transplantation. During VA-ECMO, the outflow cannula is placed in arterial circulation (13, 14).

The use of VV-ECMO in thoracic surgery is usually limited to certain procedures where adequate ventilation is not otherwise as feasible as surgery in patients with a history of previous extensive contralateral pulmonary resection, including pneumonectomy, and surgery in patients with severely compromised pulmonary function (15). Finally, VV-ECMO is the ECLS of choice for the treatment of NSCLC presenting with carinal extension requiring complex tracheo-bronchial reconstruction through an obstacle-free surgical field.

In this paper, we reviewed our experience in right tracheal sleeve pneumonectomy performed through unconventional anterolateral thoracotomy with the assistance of VV-ECMO, reporting our tips and tricks.

## Materials and methods

### Patient selection

A retrospective analysis was performed of all the consecutive patients affected by NSCLC less than 1 cm or invading carina who were referred to our institution and underwent right TSP from January 2020 to December 2022. Pathological staging was defined using the American Joint Committee on Cancer, VIII edition (16). All the patients included in this study were older than 18 years of age and were submitted to surgery after a confirmed histological diagnosis of NSCLC. All patients signed an informed consent form for the acquisition and the usage of clinical data for research purposes at admission. For the use of data, we followed the rules of the Helsinki Declaration. The study was approved by the internal research board of our centre (Humanitas Research Hospital, ID number ECMO-2023-109).

### Preoperative screening

The general performance status and the cardiopulmonary reserve were systematically evaluated during pre-treatment assessment. Pulmonary function was assessed with global spirometry: the forced expiratory volume in 1 s (FEV1) and diffusing capacity of the lung for carbon monoxide (DLCO) were evaluated. Ventilatory-perfusion assessment with pulmonary scintigraphy was obtained in all patients. Cardiac function was assessed with electrocardiography and transthoracic echocardiography.

Contrast-enhanced computer tomography (CT) of the head, chest, and abdomen as well as 18-fluorodeoxyglucose positron emission tomography (PET) were performed for the staging of all patients. The histological diagnosis was obtained through flexible bronchoscopy or CT scan lung percutaneous biopsy. The invasive mediastinal staging was performed through endobronchial ultrasound bronchoscopy (EBUS) or mediastinoscopy in case of suspected multiple metastatic mediastinal lymph node stations (2R, 4R, and 7). EBUS or mediastinoscopy were not performed when only one mediastinal station seemed to be metastatic because they only increase preoperative delay time and do not change the therapeutic strategy.

Magnetic Resonance (RM) was executed in those patients with a suspicion of mediastinal structures involvement (e.g., the heart). Instead, for the evaluation of tracheal or carinal involvement, flexible bronchoscopy was performed in all patients.

After completing all the preoperative evaluations, the treatment of every patient was discussed in our thoracic malignancies multidisciplinary team meetings. According to oncologists and radiotherapists, we usually prefer an upfront surgery even in cases of single mediastinal station involvement. The reason for this is that TSP is high-risk surgery, so upfront surgery should avoid surgical risks, especially tracheal fistula, due to the ischemic damage of the airway after neoadjuvant chemotherapy or radiotherapy.

### Surgical procedure and anaesthesia management

**Figure 1:**
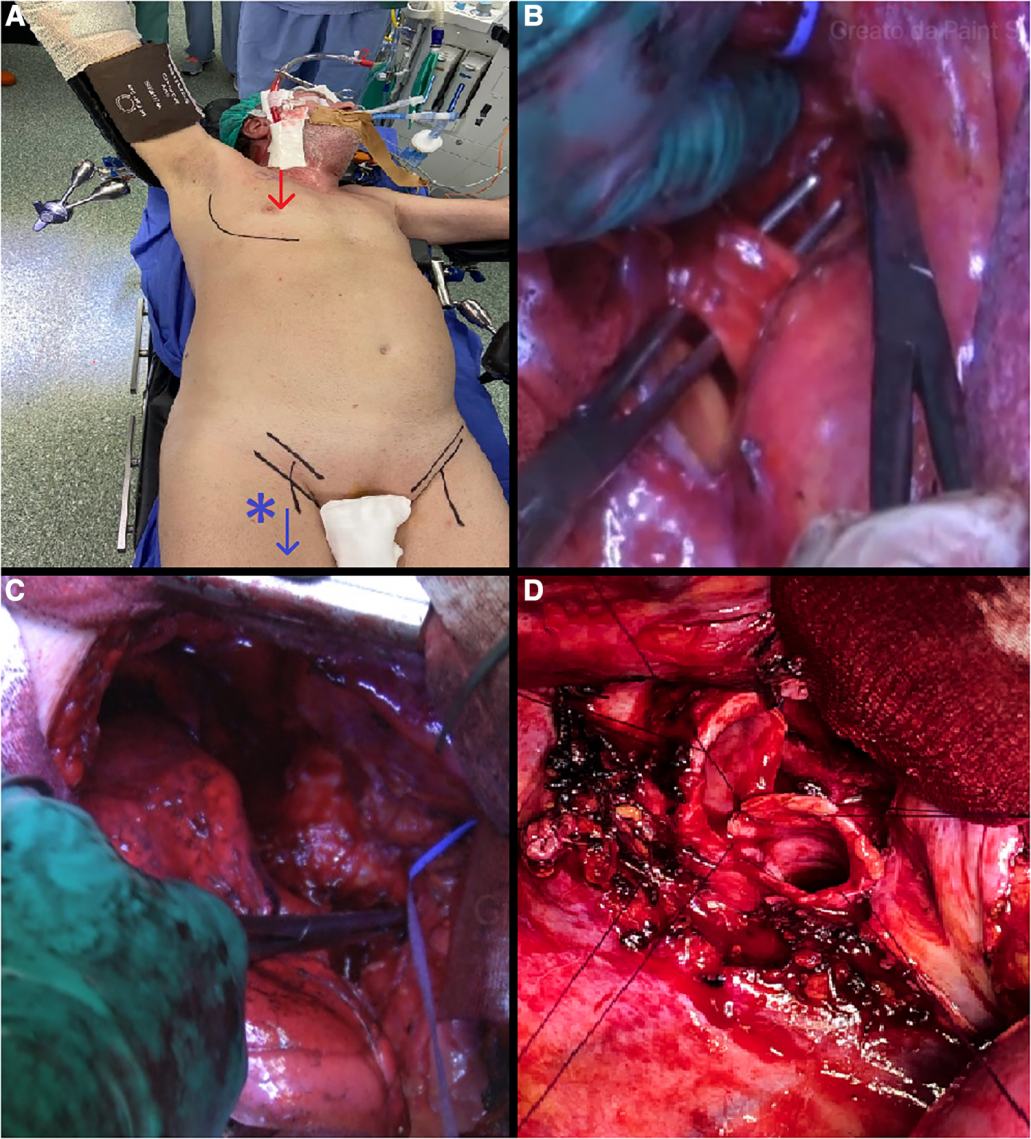
(**A**) anterolateral thoracotomy at the fourth intercostal space and VV-ECMO setting. (**B**) Isolation and division of the main right pulmonary artery at the interaorto-caval level. (**C**) Preparation and mobilisation of the trachea-bronchial bifurcation. (**D**) End-to-end trachea-bronchial anastomosis.

At the beginning of the surgery, patients were ventilated with a double-lumen tube for one-lung ventilation. We used heparin-coated percutaneous cannulas, a 16 Fr cannula was placed in the right jugular vein under ultrasound guidance, and a 24 Fr cannula was placed with percutaneous technique in the right femoral vein cannula for VV-ECMO circuit. Surgery was performed through anterolateral thoracotomy at the fourth intercostal space (Figure 1A). The first surgical step was mediastinal systematic lymph node dissection, followed by the isolation and division of the upper and lower pulmonary vein. After mobilization of the superior vena cava, a section of the Azygos vein, and incision of the pericardium, the main right pulmonary artery was usually isolated and divided at the interaorto-caval level (Figure 1B). The interaorto-caval resection of the main pulmonary artery reduces the discomfort of the stump during airway anastomosis. The tracheo-bronchial bifurcation was prepared and mobilised (Figure 1C). The limit of dissection was no more than 2 cm from the cutting level to avoid devascularization. Before completing the pneumonectomy, VV-ECMO started with a systemic heparin dose of 5,000 UI, only in the first two patients, and the pulmonary ventilation stopped. The right pneumectomy was completed, dividing the trachea 2 cm over the carina and the left main bronchus 1 cm from the origin. Airway resection could be performed with a complete section of the airway or with partial conservation of the pars membranacea. Whenever possible, preservation of the membranacea should be performed, because the more membranacea is preserved the lower the risk of anastomosis dehiscence is. End-to-end anastomosis was achieved with several interrupted 3-0 Monocryl stitches (Figure 1D). After anastomosis between the trachea and left bronchus, the double-lumen tube was withdrawn and a single-lumen endotracheal tube was placed above the anastomosis. A thymic tissue flap was placed circumferentially to wrap the anastomosis. A hydropneumatic test was performed for a first check on the anastomosis. Thereafter, the anastomosis was checked again with flexible bronchoscopy to control the bronchial lumen width and avert a too-stenotic suture. In all patients, a balanced thoracic drain (Drentech™ ANEMOS, Redax Spa, Poggio Rusco, Italy) was placed in the right thoracic cavity at the end of the surgery. The ECMO circuit was stopped after the position of the endotracheal tube. Protamine 25 mg was administrated in the first two cases to neutralize heparin action. VV-ECMO cannulas were removed at the end of surgery, and patients were extubated in the Intensive Care Unit (ICU) for 24 h if clinical conditions allowed.

In the postoperative period, pain management was guaranteed through intravenous drug administration and Erector Spinae Block (ESP). The placement of an epidural catheter was avoided because of systemic heparization.

For each patient, the following information was collected: age, sex, histological classification, induction, postoperative chemo- or radiotherapy, surgical reports, final pathology data, postoperative complications, date and modality of recurrence, and vital status. Clavien–Dindo classification was used to classify surgical complications (17).

Postoperative follow-up was carried out through 3–6 monthly clinical assessments, chest CT scans, and further *ad hoc* investigation. Follow-up data were also obtained through contact with families and general practitioners, from hospital charts, and from health registries.

### Outcome and statistical analysis

The follow-up was censored until April 2023. Overall survival (OS) was measured from the day of the surgery until death from any cause or last contact. Disease-free survival (DFS) was measured from the day of the surgery until the first recurrence. Perioperative mortality was defined as death occurring within 30 days of surgery or during the hospital stay.

A descriptive analysis was conducted to compare the preoperative, intraoperative, and postoperative features of all patients in analyses.

## Results

Three patients underwent right TSP during the study period. In all cases, we used VV-ECMO assistance for oxygenation during the anastomosis. The mean age was 56 years, and all patients had a previous smoking habit. One patient had a history of Crohn's disease, one other patient had cardio-vascular disease, and the last patient had no comorbidity. The histological diagnosis was squamous cell lung cancer in all patients. No one received neo-adjuvant chemotherapy and/or radiotherapy. Considering the clinical stage (cT2N2M0, cT3N0M0, and cT4N2M0) the main surgical indications for TSP were the presence of tumours at less than 1 cm from the carina and direct involvement of the carina. Clinical and pathological information is summarised in Table 1.

**Table 1:** Clinical and pathological patient's characteristics, ECMO details and postoperative results and complications.

ID	Age	Year	Height (cm)	Weight (Kg)	Smoke history	Comorbidity	c-Stage	Neoadjuvant therapy	Surgery time	ECMO time	Systemic heparinization	Extubating timing	Post-operative complication	p-Stage	ICU	Adjuvant therapy	Follow-up
1	55	2020	169	75	EX smoker 3 year (40/day)	Crohn	cT2N2M0 (IIIA)	NO	310 min	70 min	ACT 200’’+ protamine	5 h PO	NO	pT2bN2M0 (IIIA)	6 days	Cisplatin + vinorelbine 4 cycle	NED at 50 months
2	64	2022	180	92	EX smoker 7 years (20/day)	Hypertension, dyslipidaemia, chronic vasculopathy, ischemic heart disease	cT3N0M0 (IIB)	NO	280 min	65 min	ACT 200’’ + protamine	42 POD	FA, ARDS (MSSA, Escherichia Coli, Klebsiella Pneumoniae), tracheostomy in 15 POD, Jugular thrombosis	pT3N1 (IIIA)	42 days	–	NED at 18 months
3	49	2022	179	51	EX smoker 1 year (40/day)	NO	cT4N2M0 (IIIA)	NO	285 min	58 min	NO	1 POD	NO	pT4N2 (IIIB)	2 days	Cisplatin + vinorelbine 4 cycle	NED at 12 months

The surgical approach was systematically performed through anterior thoracotomy at the IV intercostal space (Figure 1A). Information about the surgical technique, ECMO setting, and postoperative complications and results are summed in Table 1.

Before the start of ECMO assistance, the first two patients had systemic heparinisation with activated clotting time (ACT) cut-off at 200’’ and final protamine administration. The third patient had no systemic heparinisation. The average ECMO duration was 64 min (ranging from 70 to 58 min), while the average time of surgery was 291 min (ranging from 280 to 310 min). All procedures were completed without intraoperative complications of either a surgical or anestehetic nature. The first and third patients were both extubated in the first 24 h and discharged from ICU on the 6th and 2nd postoperative day (POD), respectively, without postoperative complications. The second patient was extubated on the 42nd POD because of infective acute respiratory distress syndrome (ARDS) and underwent tracheostomy on the 15th POD. We have no experimented 30-day mortality and 90-day mortality.

All patients are alive without evidence of recurrent disease with a mean follow-up of 26.6 months.

## Discussion

Tracheal sleeve pneumonectomy is the treatment of choice for NSCLC invading the main bronchus or arising 2 cm distal from the carina, which extends to no more than three cartilaginous tracheal rings. In reporting this experience of three cases of right tracheal sleeve pneumonectomy in ECLS (VV-ECMO) we want to discuss our personal point of view concerning surgical strategy.

The surgical approach to the carina may be performed by a lateral thoracotomy, median sternotomy, or antero-lateral thoracotomy according to the surgeon's experience. Traditionally, right posterolateral thoracotomy was the most commonly used access reported in the literature (8, 9, 18, 19), although median sternotomy has been described too (20). Few surgeons published TSP procedures through antero-lateral thoracotomy (21, 22). Based on our experience, antero-lateral thoracotomy is the best option for both right and left TSP for several reasons. First, this approach allows for a good exposition of the hilum and tracheo-bronchial angle to perform both pneumonectomy and tracheal anastomosis easily. In necessary, this approach allows for rapid conversion to VA-ECMO through an easy central cannulation. Finally, in our experience, patients experience less pain compared to posterolateral thoracotomy. The patients are in supine decubitus, instead of lateral decubitus; this has several advantages concerning the possibility of preparing a single surgical field, including the femoral region. In this setting, cardiothoracic or vascular surgeons can easily and comfortably insert and manage the femoral cannula during surgery optimizing surgical time. Another advantage is avoiding cannula displacement, which could happen in lateral decubitus or during the surgical positioning of patients.

Intraoperative management of the airway is one of the main issues of this kind of surgery. The ventilation strategy must provide adequate gas exchange and oxygenation during division and anastomosis of the airway, maintains an unobstructed surgical field, and prevents blood aspiration. Different methods of intraoperative airway management have been described, including cross-field ventilation, intermittent apneic ventilation, high-frequency jet ventilation (HFJV), and extracorporeal membrane oxygenation (ECMO) (11). The ventilation of first TSPs was obtained with interrupted ventilation though the surgical field with the risk of contamination and blood inhalation (9).

In the cross-field ventilation, the surgical field visualization is disturbed due to the diameter of the tube cuff (23). The main problems were the endobronchial tube being too close to the left upper bronchus, the frequent dislocation of the tube during ventilation, blood aspiration in the lung, and ischemic damage of the main bronchus due to overinflation of the tube cuff (24) and possible infective complications due to post-operative atelectasis and less bronchial blood loss.

The HFJV is an alternative method of airway management that consists in the use of a long tube with a thin calibre and a small cuff. This method is associated with impeding complete operative exposure, prolonging surgical time due to repetitive withdrawal of the endotracheal tube, and risk of bronchial luminal injury and can also cause persistent hypercapnia, lung barotrauma, and pneumothorax (25).

ECMO assistance is usually used for severe cardiorespiratory failure management, cardiac surgery, lung transplants, and tracheal/carinal resection. The use of this technique in thoracic surgery allows hemodynamic stability with less risk for brain and myocardial oxygenation and a free operating field (13, 26, 27). On the other hand, the risk of deep venous thrombosis and surgical field bleeding can be overcome with a careful systematic heparinization controlling the ACT and the use of Protamine. Furthermore, it has been demonstrated that the risk of tumour cell diffusion is very low because the cardiotomy reservoir is absent and ECMO is a closed circulatory system starting only after significant vessel ligation (24, 28). We preferred the use of VV-ECMO because it can allow complete surgical exposure and adequate oxygenation during airway section and anastomosis; moreover, we found no complications. While in the first two cases, we used systemic heparin antagonised by protamine at the end of the procedure, for the third case we chose to not use systemic anticoagulation to decrease the risk of surgical bleeding. The reasons for avoiding anticoagulation lie in the fact that ECMO assistance time is short, and the risk of thrombosis is very low.

Another tip is about the isolation and dissection of the main pulmonary artery. No suggestion or description of the technique has been found in the literature. During TSP we systematically isolated and divided the main right pulmonary artery at the interaorto-caval level (Figure 1B). This manoeuvre can be easily performed with a good anterior exposure assured by anterior thoracotomy. The best advantage of this manoeuvre is less discomfort of the pulmonary stump and very good exposure of tracheo-bronchial angle during airway anastomosis.

The surgical technique to reconstruct the airway continuity differs between surgeons. Adsorbable material is often used to prevent granuloma formation or stenosis at the anastomosis. Weder preferred a running suture for the membranous part and interrupted sutures for the cartilaginous part with polydioxanone (PDS) 4/0 (22). Roviaro used all interrupted stitches with 3-0 polyglactin (Vicryl), knotted on the outside of the bronchus 2 mm apart on the bronchus and 3 mm apart on the trachea (8, 9). Other surgeons first performed tracheo-bronchial end-to-end anastomosis with a running 4-0 PSD suture on the deepest aspect of the airway and tied each end with independent PSD stitches, then several interrupted stiches of 3-0 PSD or Vicryl are placed in the remaining part (20, 29). Gonfiotti used the same anastomotic principles for both end-to-end and end-to-side [Barclay and Eschapasse (30, 31)] anastomoses. Interrupted stitches are usually tied with external knots when all of them have been placed to correct for size discrepancies. Our technique consists of first placing anchor stitches at the two edges and then using interrupted stitches of 3-0 Monocryl in the membranous part that are then tied. Interrupted stitches of 3-0 Monocryl are then placed on the cartilaginous part and tied at the end to control the right match of the tracheal and bronchial stump.

Although there has been development within the surgical and anaesthesiologic field, TSP remains a high-risk surgery. The main postoperative complications are (9) myocardial infarction, early respiratory failure, dehiscence of anastomosis (7), controlateral pneumonia, dysphonia, pleural empyema, and bronchopleural fistula (19, 11). The mortality rate remains quite high with a 5-year survival rate near 56% (27).

## Conclusion

Tracheal sleeve pneumonectomy remains a high-risk surgery with a quite high mortality rate. We have described safe and feasible VV-ECMO use for complex tracheo-bronchial resection, reporting our surgical tips and tricks. ECMO allows for optimal oxygenation, and at the same time it maintains an operative field that clean and calm, it favours less ischemic damage of the left main bronchus and probably contributes to reducing infective complications. This method should be reserved for centers with experience and practice in ECMO and tracheal surgery.

## Data Availability

The original contributions presented in the study are included in the article/supplementary materials, further inquiries can be directed to the corresponding author.
